# Agential Epistemic Injustice in Clinical Interactions Is Bad for Medicine

**DOI:** 10.5195/pom.2025.222

**Published:** 2025-04-10

**Authors:** Lisa Bortolotti

**Affiliations:** 1 Department of Philosophy, University of Birmingham, Birmingham, United Kingdom; Department of Neurosciences and Rehabilitation, University of Ferrara, Ferrara, Italy

## Abstract

In interactions characterized by agential epistemic injustice, the interpreter avoids engaging with the speaker’s perspective and challenges or distorts the speaker’s contribution before taking time to explore it. Where the success of the interaction depends on a genuine knowledge exchange between interpreters and speakers, epistemic injustice compromises the success of the interaction. Building on recent qualitative work on communication in youth mental health, I argue that clinical interactions are less likely to achieve their aims when practitioners fail to engage with the perspective of the person seeking support, and challenge or distort the person’s contribution before taking time to explore it.

## 1 Introduction

Since the introduction of the notion of epistemic injustice as a form of injustice that harms a person as an epistemic agent (Fricker 2007), and more than ten years of influential research on the relevance of that notion for medicine as a whole, and psychiatry in particular (for example, Carel and Kidd 2014; Crichton, Carel, and Kidd 2017), numerous studies have highlighted practices in healthcare that are at risk of perpetrating epistemic injustice. In this paper, I concentrate on a specific instance of epistemic injustice: in a clinical interaction, a healthcare practitioner challenges, distorts, or reconstrues a patient’s report of their mood and feelings, intentions, or concerns, prior to engaging with the patient about their report. In this framework, the lack of engagement with the patient’s report, and the challenge, distortion, or reconstrual of it, can be interpreted as a way for the practitioner to call into question the patient’s epistemic agency (McGlynn 2020). Epistemic agency is people’s capacity to pursue their epistemic goals, such as their capacity to acquire, produce, share, and more generally *use* knowledge. As epistemic agents are generally considered authoritative when they report how they feel, what they intend to do, or what concerns they have, the healthcare practitioner’s attitude may be the result of an assumption that a patient’s epistemic agency is compromised by their state of health.

Some of the potential harms I examine within the framework of epistemic injustice in healthcare can also be studied within alternative frameworks—for instance, by focusing on autonomy, dignity, and respect; the importance of person-centered care; and the risks of paternalism and discrimination, to name just a few. However, there are two distinctive contributions that the epistemic injustice framework makes to the literature. First, the framework of epistemic injustice may be seen as narrower than some of the alternatives, as it concentrates on how people are wronged in their capacity as *epistemic* agents. Thus, it focuses on knowledge use. Second, the framework enables us to discuss the harms that come from the person being wronged but also the harms that come from valuable knowledge being unavailable to use, within the interaction and beyond. Thus, it encourages a reflection on the costs of epistemic injustice for both parties in the interactions, as well as for society at large.

In section 2, I introduce and revisit José Medina’s notion of *agential epistemic injustice*, according to which the exercise of the epistemic agency of people from marginalized groups is constrained, manipulated, or distorted (Medina 2020, 2022). According to Medina: “Agential epistemic injustice involves the discriminatory mistreatment of the epistemic agency of members of marginalized groups; more specifically, it consists in cases in which the exercise of the epistemic agency of members of marginalized groups is unfairly constrained, manipulated, or subverted” (2022, 322).

In Medina’s formulation, agential epistemic injustice applies to members of marginalized groups, and I concentrate on a group of agents we all belong to at some point in our lives: agents playing the role of patients in a clinical interaction. Although being a patient may not be regarded as a reason for marginalization as such, recent research has highlighted the risks of pathophobia (for example, Kidd 2019) and the adverse social reactions faced by some groups of patients (for example, Blease, Carel, and Geraghty 2017).

In section 3, I offer some reasons to believe that the notion of agential epistemic injustice is well suited to characterize some of the experiences of people accessing services during a mental health crisis, because mental health conditions are often associated with agency being compromised. I offer two examples of interactions examined as part of a recent project on agency in youth mental health, and show how the framework of agential epistemic injustice can help us understand why some practices in clinical communication are problematic.

In sections 4 and 5, I defend the use of the epistemic injustice framework to analyze interactions in mental healthcare from two recent objections. First, I point out that epistemic injustice does not demand credulity. Epistemic justice demands that an interpreter engages with a speaker’s perspective prior to discounting it, when there are no reasons to doubt the speaker’s competence or reliability and where the speaker can be considered authoritative with respect to the shared knowledge. For example, the speaker’s perspective is about their own feelings, intentions, or concerns. But there are no requirements that the interpreter fully shares the speaker’s perspective after engaging with it. Second, I argue there are clinical—not merely moral—reasons to avoid epistemically unjust practices. When the practitioner challenges, reconstrues, or distorts the contribution of a person seeking support before taking time to explore it, the clinical interaction is less likely to succeed in bringing about good health outcomes for that person.

In other words, agential epistemic injustice is bad for medicine because perpetrating it as part of a clinical interaction means that key objectives of that interaction are less likely to be met. A successful clinical interaction should lead to: (a) a new or better understanding of the nature of the problem the person is seeking support for; (b) the identification of suitable means to support the person in addressing the problem; and (c) the promotion of favorable conditions for the person to address the problem via those means. In sections 5.1 and 5.2, I develop two arguments for the view that agential epistemic injustice interferes with the key objectives of clinical interactions—the *knowledge argument* and the *trust argument*. When people feel that they have not been listened to or understood, and when they have been excluded from key stages of the interaction, they are less likely to value the medical advice they receive or to reach out for further support at times of crisis.

## 2 Agential Epistemic Injustice

We tend to identify epistemic injustice with silencing or outwardly dismissing a person’s perspective. But even when no silencing occurs, and a speaker’s contribution to an interaction has been solicited, epistemic injustice can manifest in how the speaker’s contribution is received (see also Wanderer 2012 on the difference between ignoring and rejecting). *Failed uptake* can turn into challenges to the speaker’s epistemic agency. In a paper on the problems of the American judicial system, Medina identifies three kinds of agential epistemic injustice:

an epistemic injustice that results from annihilating or subverting epistemic agency …; an epistemic injustice that results from discrediting and distorting the subject’s voice to the point of nullifying or subverting her epistemic and communicative contributions …; and an epistemic injustice that results from withdrawing proper uptake and rendering the consequences of the subject’s speech acts ineffectual or self-undermining. (Medina 2020, 191)

In the forms of epistemic injustice described by Medina, a person’s testimony does not need to be silenced or outwardly dismissed for that person to be the target of epistemically unjust practices. For instance, in forced confessions, the speaker’s contribution is valued only when obtained by force, and the speaker’s agency is annihilated in the process. At other times, the speaker’s contribution is distorted to the point that they are made to say something that they did not intend to say, and their agency is nullified. Finally, the speaker’s contribution may be denied engagement and, in those circumstances, their agency loses its power.

These epistemically unjust practices do not prevent members of marginalized groups from participating in an exchange of some sort, but enable a form of participation that leads to disempowerment. There may be multiple reasons for the interpreter adopting epistemically unjust practices, but often there is an assumption that the speaker is incompetent, unreliable, or untrustworthy. This assumption is triggered by aspects of the speaker’s identity that negatively affect the interpreter’s view of the capacity of the speaker to exercise their epistemic agency.

In one form of agential epistemic injustice, *contributory injustice*, the speaker is thought to be unable to make valuable contributions to shared epistemic projects, and thus their original contribution is reconstrued as something different from what they intended (Hookway 2010). In Christopher Hookway’s example, a student from a marginalized group raises an objection, which the lecturer treats as a request for clarification. Notice here that not only is the student wronged as an epistemic agent in this circumstance, but the quality of the whole class discussion is also negatively affected by the lecturer’s behavior. If the lecturer aims at fostering a good discussion and developing the students’ argumentative skills, then failing to engage with interesting objections is an obstacle to the achievement of those goals.

A case of agential epistemic injustice that involves distortion is *harmful inclusion*. A person is asked to contribute epistemic labor to fill gaps in societal understandings of exclusion and discrimination. But the contribution does not enable the person to adequately characterize the problems from the point of view of a marginalized individual or the member of a marginalized group (Pohlhaus 2020). For instance, burdening members of marginalized groups with the need to provide evidence for discrimination in ways that satisfy the demands of the dominant culture might mean that microaggressions are not addressed because they cannot be evidenced to the required standards. Notice here that, if the goal of the exercise is to achieve progress with equality and inclusion, the constraints imposed on the contributions by members from marginalized groups may be an obstacle to the achievement of those goals. In some forms of harmful inclusion, the speaker’s contributions are constrained. Think about how expertise by experience is integrated with other forms of expertise—for instance, when the contribution of people with lived experience is restricted to certain tasks and does not have the general applicability of contributions by other experts (for example, Woods, Hart, and Spandler 2022; Okoroji et al. 2023; Larkin, Bortolotti, and Lim 2024).

When reflecting on the cases of agential epistemic injustice discussed in the literature, it is striking that when the speaker’s contribution is challenged, reconstrued, constrained, or distorted, the success of interactions that require a genuine knowledge exchange is under threat. In the rest of this paper, I focus on mental healthcare interactions and argue that epistemically unjust practices can be an obstacle to the success of such interactions.

## 3 Agential Epistemic Injustice in Mental Healthcare

The notion of agential epistemic injustice is especially apt to capture problems with interactions involving people who are experiencing a mental health crisis because the stigma associated with poor mental health tends to target people’s epistemic agency. When people struggle with their mental health, it is assumed that their agency is compromised, and their capacities for rational thought, appropriate affect and mood, competent decision-making, and autonomous action (among other relevant capacities) are negatively affected by their health condition. According to Karen Newbigging and Julie Ridley: “People experiencing mental distress are particularly vulnerable to epistemic injustices as a consequence of deeply embedded social stigma resulting in a priori assumptions of irrationality and unreliability such that their knowledge is often discounted or downgraded” (2018, 36).

In the case of serious mental illness, the assumption often is that the capacities to acquire, produce, share, and use knowledge, and more generally to behave in agentic ways, are *severely* compromised, and this allegedly justifies disengagement (Dorfman and Reynolds 2023), or pervasive forms of exclusion (Ritunnano 2022) and coercion (Spencer 2023) that would not be acceptable if those capacities were thought to be intact. As shown in several studies (for example, Grim et al. 2019; Byrne et al. 2021; Bergen et al. 2022), mental health patients may face the following situations in clinical interactions: being devalued as testifiers, experiencing clinical communication as a confrontation, having testimonies edited or challenged, and being excluded from key aspects of the interaction, such as the discussion of the diagnosis or the choice of treatment.

To some extent, all patients play a subordinate and passive role in interactions with medical professionals—they are seeking support, and healthcare practitioners have the competence and authority to offer support because of their medical expertise and clinical experience. But the mental health patient may be considered at an increased risk of epistemic injustice. In a mental health crisis, even people’s reports of their experiences and concerns can be challenged, and this is significant because agents can usually claim some authority over their thoughts, feelings, and intentions. Mental health patients may be regarded as unable to understand whether their experiences are problematic, whether their concerns are legitimate, and to what extent they are responsible for their own ill-health. Not only are they subordinate to healthcare practitioners in the power dynamics of the relationship, as is any other patient; there is also a heightened risk that their reports and concerns are pathologized and seen as a manifestation of their condition—for example, in the case of dementia (Chattat, Trolese, and Chirico 2025) and in the case of hearing voices (Bortolotti et al. 2025). Mental health patients may be deemed too ill to accept a diagnostic label as appropriate, or to agree on proposed means of support or treatment for the future. As a result, their collaboration and agreement on such issues are not always sought or valued.

In the rest of this section, I apply the notion of agential epistemic injustice to two examples of mental healthcare interactions that were considered problematic when examined by triangulating evidence from conversation analysis on video-recorded liaison psychiatry assessments and follow-up interviews with participants. Does agential epistemic injustice adequately explain why the interactions were deemed unsuccessful? The aim of the examples is not to show that agential epistemic injustice is prevalent, or even frequent, in mental healthcare interactions. We need custom-made empirical data to support that conclusion. Rather, the aim is to suggest that, when practices in clinical communication raise concerns, the notion of agential epistemic injustice might help us understand the nature of those concerns and identify remedies to those practices.

The two examples are based on a recent qualitative study of video-recorded interactions between mental healthcare practitioners and young people accessing emergency mental health services in the United Kingdom for problems with their mood, suicidal thoughts, and self-harm (Bergen et al. 2022, 2023). The interactions have been analyzed by a team of researchers, including sociologists, psychologists, clinicians, philosophers, and lived experience researchers. Two team members who specialize in conversation analysis examined the interactions. Conversation analysis is a method developed in sociology (Sacks, Schegloff, and Jefferson 1974) that studies how speakers and listeners make sense of each other’s contributions in an exchange. Such a method considers *what* people say and *how* they say it, including an analysis of prosody and embodied communication. In the extracts I reproduce here, the perspectives and concerns of the young people accessing services are not thoroughly explored but are challenged or reconstrued by the practitioners—or, at least, this is the conclusion the research team reached, based on the recordings of the interactions and on the young people’s post-assessment interviews:

When people’s experiences of suicidality and self-harm were not accepted or were undermined, questioners did not acknowledge or accept the person’s account; asked questions that implied inconsistency or implausibility; juxtaposed contrasting information to undermine the person’s account; asked questions asserting that, e.g., asking for help implied they were not intending to end their life; and resisted or directly questioned the person’s account … Alternative characterizations were used to justify decisions not to provide further support or referrals to specialist services. At times, these practices were also delivered when speaking over the patient. (Bergen et al. 2023)

In the first case, Robert is referred to emergency services from university counselors.^1^ He is asked how he feels during the interaction and initially describes himself as being “miserable” and feeling “suicidal.” Robert’s description is challenged by the healthcare practitioner, who suggests that Robert does not always look miserable and, given that he had plans to meet some friends that night, he did not genuinely entertain thoughts of ending his life. Here is a brief extract of the recorded interaction between Robert (PT) and the practitioner (PR):

1 *PT: So I (.) feel like miserable kind of (.) sums it up*,
2 *PR: And yet in your fa:ce, you [know=*
3 *PT:                 [Yeah*,
4 *PR: =when you’re speaking. You’ve You’ve got a variation. haven’t*
5 *you. of- of your expressio:n,=and you know you smi:le and*
6 *things like that*.
7 *PT: >Yeah,< ((no nonverbal response))*
8 *PR: >So you have times< when you clea:rly (0.3) aren’t miserable*,
9 *you’re sort of enjoying things, or you’re able to [give the*
10 *PT: [Mhm*,
11 *PR: impression [that you are enjoying thi:ngs*,
12 *PT:                 [Yeah, ((small nod))*
  …
47 *PR: What- What plan would you have [had if you*
48 *PT: [I just- Well I’ve got a*
49 *few events on. ‘Cause I’m part of rugby skiing and tennis*.
50 *And they were all putting events on tonight I couldn’t go*
51 *to*.
52 *PR: I see. So could we safely say, you know. you wouldn’t end*
53 *your life?*
54 *(1.0)*
55 *PR: Or something that would have=*
56 *PT: =What tonight?*
57 *PR: Yeah. [Y*
58 *PT: [I wouldn’t have ended it toni:ght. ((shakes head))*
59 *PR: ((nods)) You wouldn’t have. Okay. So maybe there was a bit*
60 *of miscommunication because they they brought you he:re*
61 *because they were saying you were suicida:l, and=*
62 *PT: =No I ((nod)) am.=But [I*
63 *PR:                 [You a:re*.
64 *PT: But I’ve- I feel I can (3.0) I mean I haven’t done it yet*,
65 *PR: Mm. ((nods))*
  (Bergen et al. 2023)

Robert is asked about his mood and intentions by the practitioner and gets to talk about how he feels and what he meant to do. However, Robert’s replies are challenged by the practitioner before there is an opportunity to explore why Robert says that he feels miserable and suicidal. There is also a suggestion that Robert might not have been unwell enough to visit emergency services. At the end of the interaction (which does not appear in the extract above), Robert is advised to visit a self-help website and continue accessing university counseling. No further support is offered.

In the second case, Gemma accesses emergency services due to the symptoms of obsessive-compulsive disorder (OCD) worsening and involving self-harm and suicidal thoughts. But it becomes clear during the interaction that she is mostly worried about her calorie intake. Gemma feels that her OCD requires her not to assume more than 800 calories a day. She claims to have lost a lot of weight and is now close to being underweight. The practitioner (PR) downplays the eating problem reported by Gemma (PT).

1 *PR: And and in terms of you:r understanding. What’s your diagnosis*
  …
3 *PT: Um: OCD, and (.) anxiety, I think, ((shakes head))*
4 *PR: Okay. ((nods))*
5 *PT: ((nods))*
6 *PR: And you- That- For you: that makes sense does it. ((nod))*
7 *PT: Yes. ((nod)) The only thing that doesn’t make sense is why:(.)*
8 *I’m feeling unable to eat:. [And restricting what I’m eating*.
9 *PR:                 [Mm::. ((nod))*
10 *PR: Okay*.
11 *PT: And having (.) um (.) ((voice breaks)) kind of unpleasant*
12 *thoughts about my body shape? [and*,
13 *PR:                 [Mm:. ((nod)) Okay*.
14 *PT: that*.
  …
15 *PR: Alright, Okay, And I assume that you’re rea:lly (.) try:ing?*
16 *eating, ((nod)) as in you’re (.) you know trying to give*
17 *yourself permission (.) to (.) you know, enjoy food. Whatever*.
18 *(.) ‘Cause I guess if you’re quite slim and you’re worried*
19 *about losing more weight. Now’s not ((shakes head)) the time*
20 *to start thinking Well I shouldn’t have any custard ((smiles))*
21 *or I [shouldn’t have any- So you’re trying t- Are you trying to*
22 *PT: [((looking down, nods)) °Mm.°*
23 *PR: just have what you fa-fancy when you-when you could (.) eat*
24 *it*.
25 *PT: I- ((shakes head))*
26 *PR: Again it’s e:asier said than [done but*,
27 *PT:                 [Whatever it is it’s not letting*
28 *me*.
29 *PR: It’s not what, [Sorry*.
30 *PT:                 [It’s not letting me*.
31 *PR: Right*.
32 *(2.0)*
33 *PR: Okay*.
34 *PT: Like I- (1.0) haven’t eaten anything today*,
35 *PR: Mm*.
36 *PT: And I’ve barely eaten anything since Monday, [Just*
37 *PR:                 [Okay*.
38 *PT: Yeah. It’s got out- out of control*.
39 *PR: Mm::. Okay, ((nods, looks away))*
40 *(1.0)*
  (Bergen et al. 2023)

In the exchange, there is ample opportunity for Gemma to talk about her eating problem, but there is a marked difference between the way Gemma talks about it as something that she cannot control and the way the practitioner talks about it as something that can be feasibly managed without additional intervention. Gemma refers to eating as something that she cannot do (“I’m feeling unable to eat,” “it’s not letting me,” “It’s got out- out of control”) and describes it as a serious problem that raises concerns in herself and the people around her. The practitioner does not substantially engage with such concerns and their contributions and their line of questioning suggests instead that it is in Gemma’s power to eat more (“just have what you fa-fancy when you-when you could (.) eat it”). Later in the interaction, when it is time for the practitioner to propose means of support for Gemma, the practitioner suggests that Gemma should ask someone to sit with her at mealtimes. Whereas Gemma would like to get specialist support with her eating, for the practitioner self-help and support from friends and family are sufficient at this stage.

In the two extracts considered here, the young people participate in the exchange and have an opportunity to both describe how they feel and articulate their concerns. But their reports are challenged and reconstrued according to the practitioner’s understanding of their situation before they can be validated and explored. Statements that support the practitioner’s view are accepted and integrated in a narrative explanation of the situation that goes on to inform the decision-making process, such as Robert’s mentioning that he would have not ended his life that very night. But statements that are in tension with the practitioner’s view are ignored or undermined, such as Gemma’s emphasis on eating restrictions not being under her control. Gentle pressure is exerted via rephrasing, repetition, and close questioning. As Clara Bergen and her colleagues (2023) notice, the style of questioning is at times forensic—the practitioner seemingly attempts to identify inconsistencies in the person’s spontaneous reports.

## 4 Epistemic Justice Versus Credulity

The notion of agential epistemic injustice is useful as an interpretive lens on communication in mental healthcare interactions: it makes epistemic agency salient to the exchange, and helps us understand that, while a minimal form of speaker’s participation may be enabled and even encouraged, the speaker’s contribution is received in ways that question their competence and reliability—for instance, their capacity to describe accurately how they feel and their capacity to identify reasons for concern.

However, not everybody agrees that epistemic injustice is the right lens to use in the interpretation of mental healthcare interactions. Recently, Brent M. Kious, Benjamin R. Lewis, and Scott Y.H. Kim (2023) have suggested that it is unreasonable to expect mental healthcare practitioners to *always believe* what people seeking support say, and that the demands of epistemic justice may be *clinically unhelpful*, pushing practitioners to behave in ways that do not promote positive health outcomes for the people seeking support:

We may … have frank disagreements with proponents of EI [epistemic injustice] about when psychiatrists are obligated to “believe” their patients. Suppose we are consulting in the ER on a patient who is suicidal and depressed. He denies having made any suicide attempt in recent days, but we note that his acetaminophen level is nearly toxic and that there are circumferential abrasions on his neck that suggest a recent hanging attempt. We’d initially be inclined to doubt his testimony, and to look for additional information (alternative explanations for the abrasions) to help us discern whether his denial is accurate. We worry that many advocates for epistemic justice would regard this as morally wrong, and say we should simply take the patient at his word. (Kious, Lewis, and Kim 2023, 3)

I shall briefly deal with the first objection here and address the second objection in more detail in the next section, at it lies at the core of my argument in this paper. Minimally, epistemic justice is absence of prejudice. It does not demand that an interpreter always believe or never doubt the speaker’s testimony, nor does it demand that the interpreter share the same explanation or interpretation of the facts offered by the speaker. Epistemic justice does demand, however, that the way in which the interpreter receives the speaker’s testimony is not affected by prejudice. Unless the interpreter has prior evidence that the speaker is incompetent, unreliable, uncooperative, or deceitful, an epistemically just interaction should be characterized by genuine engagement, where both parties share their perspectives and consider the other person’s perspective with an open mind.

In particular, the notion of *agential* epistemic injustice I have applied here is about the assumptions an interpreter makes about the capacity of the speaker to acquire, produce, share, and use knowledge. If the interpreter assumes that the speaker is incompetent, unreliable, uncooperative, or deceitful without good reason, there are grounds for epistemic injustice. But the fact that the interpreter does not share the speaker’s perspective does not count as evidence of epistemic injustice. As others have already observed, epistemic injustice neither demands credulity (Kidd, Spencer, and Harris 2023), nor is it antithetical to evidence-based reasoning (Radoilska and Foreman 2023).

The problem in the extracts considered here is not that the practitioner fails to believe the young person’s report, or comes to a different interpretation of the young person’s concerns. No epistemic injustice needs to be involved if the practitioner’s attitude is based on prior engagement with the person’s perspective. Epistemic injustice may be involved if, prior to a meaningful engagement with the person’s reports of their experiences and concerns, the practitioner challenges such reports, suggesting that they are a misrepresentation or an exaggeration, and implies that the experiences the person is concerned about are not worth the attention of emergency services.

Robert offered his own understanding of how he felt (“I am miserable”) and Gemma shared her main health concern (“I’m feeling unable to eat”), and the response of the practitioner in both cases was to reframe their contributions into something else (“And yet in your fa:ce, you [know … when you’re speaking. You’ve You’ve got a variation. haven’t you. of-of your expressio:n,=and you know you smi:le and things like that”; “trying to give yourself permission (.) to (.) you know, enjoy food”), without Robert’s and Gemma’s perspectives being explored first. It is significant that Robert and Gemma were challenged on reports that interpreters typically take speakers to be authoritative about; that is, reports about the speaker’s own feelings and experiences. Of course, speakers are not infallible when they describe their own feelings and recount their own experiences, and it is possible that they exaggerate how miserable they are when they are having distressing thoughts, or how difficult it is for them to eat when they feel that restrictions are imposed on them by their OCD. But the bar for challenging those reports should be higher than for challenging reports of a different nature, where margins for errors are greater. If there is one area in which speakers can claim some expertise, it is how things seem and feel *to them*. It is noticeable that in the extracts the practitioners showed almost no engagement with the reports and showed no interest in finding out why Robert and Gemma described their mood and predicament in the way they did. An exploration of the person’s perspective does not imply acceptance, as it has been argued in a number of contexts, such as depression (Pies 2013) and chronic fatigue syndrome (Byrne 2020). It may involve encouraging the speaker to say more, and showing some curiosity about what the speaker’s view is, and what might have brought it about (Bortolotti and Murphy-Hollies 2023).

By contrast with the two examples we have discussed, in the examples of successful interactions examined by Bergen and colleagues (2022, 2023), practitioners explore young people’s reports, asking questions and showing an interest in the events preceding the crisis. Then, practitioners analyze possible causes, together with the young people, before settling on an interpretation. Independent of whether an agreement can be reached in the end, the young person feels listened to and their concerns are not delegitimized.

So, why do practitioners sometimes fail to engage in this way? There are several possible answers to this question, and it is difficult to adjudicate between them. One possibility is that, as a result of their accessing emergency services for a mental health crisis, young people are not always seen at the time as competent and reliable agents capable of sharing information that is worth taking on board. A more charitable explanation is that sometimes practitioners attempt to reframe the problems in a way that would keep the young person safe and “help them to help themselves” in a context where demand for specialist support is high and resources are limited. Independent of what motivates the practitioners’ communication style, one undesirable consequence is that the interaction does not make young people feel that they have been listened to, and limited engagement might prevent valuable information about the young people’s experiences from being shared.

## 5 Clinical Reasons to Avoid Epistemic Injustice

Is epistemic justice clinically irrelevant or even unhelpful when we are concerned with improving health outcomes? It is not inconceivable that in some contexts the moral demands of epistemic justice might pull in a different direction from the demands of good clinical practice and that genuine dilemmas may arise. However, here I argue that practices that amount to challenging and reconstruing or distorting people’s contributions, before taking time to explore them, are an obstacle to successful clinical interactions. In particular, the claim is that epistemically unjust practices are likely to make clinical interactions less likely to be successful when measured against the key goals that interactions of that type are supposed to achieve.

We may consider a clinical interaction successful when it leads to: (a) understanding the nature of the problem the person is seeking support for; (b) identifying the best means of support for the person experiencing the problem; and (c) promoting favorable conditions for the person to address the problem via those means. There is a wide recognition in the literature that good communication is central to ensuring that clinical interactions achieve such goals: “Effective communication facilitates the accuracy of information transmitted in the clinical interaction, contributes to reducing stress and offering support to patients, and fosters patients’ active involvement in their care” (Martin and DiMatteo 2013, 833).

Sections 5.1 and 5.2 develop two arguments for the view that agential epistemic injustice interferes with the pursuit of the key objectives of clinical interactions. According to the *knowledge argument*, behaviors that compromise the knowledge exchange between the practitioner and the person seeking support can be an obstacle to an adequate understanding of the problem and to a well-informed choice of means of support. According to the *trust argument*, behaviors that undermine—rather than affirm—the agency of the person seeking support can be an obstacle to the person developing trust in their capacity to contribute to positive outcomes and trust in services to provide adequate support.

### 5.1 The Knowledge Argument

A genuine exchange of information between a healthcare professional and a person who seeks support enables knowledge about the person’s experiences to be shared, including knowledge that the person themselves has better access to, such as how a mental health issue is affecting their life. When this knowledge is shared, it feeds into the process by which the problem is identified, and a diagnostic label is used to describe it. It also affects the choice of support or treatment that can be made during the interaction. Agential epistemic injustice may prevent a genuine exchange of information from happening, if people seeking support share their experiences but healthcare practitioners fail to engage with such reports, and challenge or distort them before exploring them further. This potentially leads to practitioners offering a premature diagnostic label, or suggesting support and treatment options that are based on partial or distorted information, and thus may turn out to be misguided or ineffective.

The opportunity for the person who seeks support to genuinely contribute to the interaction and participate in decision-making processes enables the practitioner to propose means of support and treatment options that better address the person’s concerns. In an interaction characterized by agential epistemic injustice, the person may express their concerns but receive no uptake, or refrain from expressing their concerns for fear that these will be discounted. Concerns may be reinterpreted in the light of the belief that the person is “being difficult,” or that “it is their illness speaking.” This potentially leads to a choice of support or treatment that does not meet the person’s needs.

Enabling the person to ask questions and share concerns has important psychological and communicative functions in the context of the interaction, but it also furthers the clinical aims of the interaction. First, the person seeking support feels more involved in decisions about their health and has a chance to obtain information about the medication or forms of support proposed by the practitioner. This is useful in managing their expectations and can contribute to better adherence. In addition, when the person shares their perspective without fear of judgment, the information can contribute to the practitioner’s understanding of the extent to which the proposed medication or form of support is likely to address the specific needs of that particular person—as opposed to the needs of people who present with similar problems or have the same diagnosis. Exploring the person’s perspective may alert the practitioner to existing problems and prevent future nonadherence, as in this example from a qualitative study by Karin Bacha, Terry Hanley, and Laura Anne Winter:

When practitioners understood and put the participants’ needs at the center of decisions about their care, the participants said the treatments provided were more effective. Fayah described her experience with a helpful inpatient ward psychiatrist:

“He [ward psychiatrist] listened to what I was saying. He took everything very slowly. He went at my pace, listened to my concerns and to any worries I had about side effects. If I wanted to come off a medication because of the side effects he was there. If I needed help with sleep, he’d say ‘right we’ll find a way around to help you’.”

Fayah explained that the psychiatrist’s actions of listening, letting her lead the pace of treatment, and acknowledging her worries about the side effects of treatments effectively helped to stabilize her symptoms, resulting in an earlier than planned discharge from hospital and the sustained stabilization of her mental health difficulties. (Bacha, Hanley, and Winter 2020, 378)

In sum, key goals (a) and (b) of the clinical interactions—that is, understanding the nature of the problem the person is seeking support for and identifying the best means of support for the person experiencing the problem—are less likely to be met when agential epistemic injustice takes place. In the next section, I shall argue that agential epistemic injustice also compromises goal (c) of the clinical interaction—that is, promoting favorable conditions so that the person seeking help can address their problem via the means recommended by the healthcare professionals.

### 5.2 The Trust Argument

As part of the study on clinical communication in interactions with young people accessing emergency services (Bergen et al. 2022, 2023), there was a follow-up interview with the young people whose interactions were recorded, immediately after the interaction, and three months after their visit to the emergency services. We know that Robert returned to the emergency department on two separate occasions, for suicidal thoughts and for an overdose. In an interview that took place three months after the initial visit, Robert said that he would have not visited the emergency department again but was told to do so by the counseling services at his university (Bergen et al. 2023). In an interview that took place three months after her initial visit to the emergency department, Gemma said that her impression was that she had not been taken seriously because she did not look underweight at the time, but she had lost a lot more weight since then, qualifying now for anorexia. She also added that, if she had accessed specialist help earlier, maybe her condition would have not worsened so fast (Bergen et al. 2023).

We can infer from the post-assessment interview data that their initial visits to the emergency services were not particularly good experiences for Robert and Gemma. The young people did not feel that they were listened to and, at least in Robert’s case, they would not have gone back to the same services if they could have avoided it. There are moral reasons for practitioners to engage with the reports of people seeking support and to ensure genuine participation: it is important to make people feel that their perspective is valued, especially when they are vulnerable and experiencing a crisis (for example, they are brought to emergency services because they are feeling suicidal). But another argument for avoiding epistemically unjust interactions in healthcare is that they are an obstacle to the interaction playing a role in promoting better health outcomes. Changes to communication style might not have prevented Robert’s and Gemma’s mental health from deteriorating further in the months following their visits, but might have contributed to the young people developing trust in the capacity of services to support them: “These findings have important clinical implications: patients report that when their experiences are not accepted or undermined, this makes them more distressed, less hopeful about the future and discourages future help-seeking when in crisis” (Bergen et al. 2023).

The observation by Bergen and her colleagues is confirmed by existing literature on the connection between trust in the therapeutic relationship and health outcomes (for example, Birkhäuer et al. 2017; Stubbe 2016). Interactions where people’s agency is questioned and undermined are not conducive to mutual trust, whereas supportive social interactions have a powerful effect on a person’s sense of agency. There are two dimensions of trust that are relevant here: trusting oneself and trusting the other, where trustworthiness is linked to perceptions of competence and benevolence (Palafox-Harris 2025).

Let us start with trusting oneself. Clinical interactions where people feel that they are listened to and understood, and where their contribution is solicited and engaged with, strengthen the sense that they are competent agents whose participation in shared epistemic projects is valued by others (Rogers 2002). This “boost to agency” may translate into goal pursuit and goal achievement. It consists of cognitive and motivational components, which involve people perceiving themselves as capable of pursuing their goals and perceiving their goals as desirable and achievable, without downplaying existing challenges, but developing the confidence to face those challenges, with adequate support (Bortolotti 2018). Illness undermines people’s sense of competent agency by affecting both whether people represent their goals as desirable and achievable and whether they represent themselves as capable to pursue them. When people are unwell and seek help to regain their health, illness typically makes it harder, or even impossible, for them to pursue some of their goals. For instance, a serious injury compromises mobility and self-sufficiency, engendering the feeling that the person has become weak and a burden to others. Being diagnosed with a chronic condition or acquiring a permanent disability can give rise to hopelessness and a sense of loss. For this reason, in some sociological accounts, illness is a described as a disruption to people’s biographies (Bury 1982).

When people experience a mental health crisis, the threat to their sense of agency is magnified. This is due to the nature of their symptoms and the self-doubt and stigma triggered by adverse reactions to mental health conditions in social contexts (Lysaker and Leonhardt 2012; Houlders, Bortolotti, and Broome 2021). Low mood, unusual experiences, and paranoid thoughts can affect the way people see the world, presenting it as often very different from how they are used to seeing it, and less predictable. Distressing experiences can make people feel helpless, as if they were losing control not just of their environment but also of themselves. Feeling that they are not listened to or understood, or experiencing exclusion from shared epistemic projects in social interactions, as a result of being perceived as incompetent or unreliable, can further undermine a sense of agency that is already under threat.

Assuming the role of patient often is a cause of disempowerment. In clinical interactions, people’s epistemic agency may be questioned or downgraded merely due to their being ill, as Havi Carel and Ian James Kidd notice, even if there are no objective reasons for their testimonies to be challenged: “An ill person may be regarded as cognitively unreliable, emotionally compromised, existentially unstable or otherwise epistemically unreliable in a way that renders their testimonies and interpretations suspect simply by virtue of their status as an ill person with little sensitivity to their factual condition and state of mind” (Carel and Kidd 2014, 530).

As we saw in the previous sections, in interactions with emergency services for suicidal thoughts, depression, and self-harm, young people’s testimonies can be challenged, distorted, or reconstrued. In the context of other mental health conditions, such as psychosis (Arboleya-Faedo et al. 2023), personality disorders (Klein, Fairweather, and Lawn 2022), and dementia (Jongsma and Schweda 2018), people describe experiences in clinical encounters as infantilizing and dehumanizing, and report being treated like young children or animals who cannot make decisions for themselves and need to be in the care of others because they do not know what is good for them. In such cases, patients’ experiences and concerns are not considered as valuable contributions to the exchange, and patients are unlikely to play an active role in the processes of arriving at a diagnosis or choosing appropriate means of support. This may contribute to people feeling incompetent and losing trust in themselves.

Let us now move to the second dimension of trust, trusting the other. The quality of social interactions affects participants’ capacity to develop mutual trust. When a person seeking support feels that they are listened to and understood, they have their sense of agency safeguarded and are more likely to develop a relationship of trust with practitioners. This sense of inhabiting a safe space persists even when the perspective of the practitioner does not match the person’s perspective in the end. If people seeking support feel that the practitioner gains sufficient knowledge of the relevant factors in the crisis they are experiencing, and engages with them as persons with complex needs and interests, the practitioner earns their trust by satisfying both the competence and the benevolence criteria for trustworthiness. As a result, people seeking support are better placed to make a positive contribution to their health journey by taking ownership of the medical advice they receive and reaching out for further support at times of crisis.

When interactions are characterized by agential epistemic injustice, people seeking support can perceive lack of engagement or challenges to their own reports as a sign that their concerns are delegitimized and there is no genuine attempt to understand what is happening to them. If the practitioner treats them just as a diagnostic puzzle to solve, this may suggest that the practitioner is not interested in what they are experiencing or is unwilling to explore their concerns further. Medical advice is less likely to be perceived as something worth following and, if another crisis is experienced, people may be reluctant to seeking support again for fear that their reports will be challenged and their concerns delegitimized once more:

In response to feeling threatened, disempowered, unsafe, and vulnerable in mental health services, the participants lied, became passive or disengaged from mental health practitioners to regain a sense of control and protect themselves. Marcus stated: “If you don’t get empathy from someone, then you’re not going to work with them.” (Bacha, Hanley, and Winter 2020, 376)

Absence of trust in oneself and in the practitioner interferes with the third goal of the clinical interaction; that is, the promotion of an environment that is conducive to the person making a positive contribution to restoring or improving their health.

## 6 Conclusions

In this paper I have argued that if healthcare practitioners do not engage with the contributions of people seeking support in the course of clinical interactions, by exploring patient perspectives before challenging or reinterpreting them, they may not be able to access valuable knowledge about people’s experiences that could inform diagnostic processes and the selection of adequate means of support.

Further, if healthcare practitioners do not acknowledge that people seeking support are agents with a valuable perspective to share and with the capacity to contribute to positive change and to participate in decision-making, people’s sense of agency may be threatened not just by the nature of their mental health issues and their role as patients, but also by the quality of the clinical interactions they experience. This may undermine people’s motivation to trust themselves as competent agents and to trust healthcare professionals, with negative implications for their likelihood to follow medical advice, seek further support when needed, and adhere to medication.

When the interpreter challenges, distorts, or reconstrues the speaker’s contribution, before taking time to explore it, this can compromise the capacity of that interaction to fulfill its goals. This is because, for some interactions to be successful, all parties need to be able to make a genuine contribution that is valued. Clinical interactions are interactions of that type, and this explains why it is a mistake to interpret concerns about epistemic injustice in medical practice as yet another demand to be made of healthcare professionals, something that may distract them from their “real job,” or even undermine their attempts to improve the health outcomes of people seeking support. In interactions characterized by asymmetrical power relations—in the home, in the classroom, and in the workplace—where the success of the interactions lies in all participants making a genuine epistemic contribution, practices characterized by agential epistemic injustice are an obstacle to the success of the interactions. Clinical interactions are no different.

## Footnotes

1 For research ethics reasons, the names of the participants have been changed.
